# Peanut diversity and specific activity are the dominant IgE characteristics for effector cell activation in children

**DOI:** 10.1016/j.jaci.2021.02.029

**Published:** 2021-08

**Authors:** Oliver Hemmings, Umar Niazi, Matthew Kwok, Louisa K. James, Gideon Lack, Alexandra F. Santos

**Affiliations:** a Department of Women and Children’s Health (Pediatric Allergy), School of Life Course Sciences, Faculty of Life Sciences and Medicine, King’s College London, London, United Kingdom; b Peter Gorer Department of Immunobiology, School of Immunology and Microbial Sciences, King’s College London, London, United Kingdom; c Asthma UK Centre in Allergic Mechanisms of Asthma, London, United Kingdom; d Guy’s and St Thomas’ National Health Service Foundation Trust and King’s College London National Institute for Health Research Biomedical Research Centre Translational Bioinformatics Platform, Guy’s Hospital, London, United Kingdom; e Blizard Institute, Queen Mary University of London, London, United Kingdom; f Children’s Allergy Service, Evelina London Children’s Hospital, Guy’s and St Thomas’ Hospital, London, United Kingdom

**Keywords:** Avidity, diversity, specificity, specific activity, specific IgE, basophil activation test, mast cell activation test, peanut allergy, CD63, AUC, Area under the curve, ISAC, Immuno Solid-phase Allergen Chip, LAD2, Laboratory of Allergic Diseases 2, MAT, Mast cell activation test, NA, Nonsensitized nonallergic, PA, Peanut-allergic, PD, Peanut diversity, PS, Peanut-sensitized but tolerant, ROC, Receiver-operating characteristic, SA, Specific activity, sIgE, Specific IgE, SPT, Skin prick test, tIgE, Total IgE, WD, Whole diversity

## Abstract

**Background:**

IgE mediates allergic reactions to peanut; however, peanut-specific IgE (sIgE) levels do not always equate to clinical peanut allergy. Qualitative differences between sIgE of peanut-sensitized but tolerant (PS) and peanut-allergic (PA) individuals may be important.

**Objective:**

We sought to assess the influence of IgE characteristics on effector cell activation in peanut allergy.

**Methods:**

A cohort of 100 children was studied. The levels of IgE to peanut and peanut components were measured. Specific activity (SA) was estimated as the ratio of allergen-sIgE to total IgE. Avidity was measured by ImmunoCAP with sodium thiocyanate. IgE diversity was calculated on the basis of ImmunoCAP-Immuno Solid-phase Allergen Chip assays for 112 allergens or for 6 peanut allergens. Whole-blood basophils and mast cell line Laboratory of Allergic Diseases 2 sensitized with patients’ plasma were stimulated with peanut or controls and assessed by flow cytometry.

**Results:**

SA to peanut (*P* < .001), Ara h 1 (*P* = .004), Ara h 2 (*P* < .001), Ara h 3 (*P* = .02), and Ara h 6 (*P* < .001) and the avidity of peanut-sIgE (*P* < .001) were higher in PA than in PS individuals. Diversity for peanut allergens was greater in PA individuals (*P* < .001). All IgE characteristics were correlated with basophil and mast cell activation. Peanut SA (*R* = 0.447) and peanut diversity (R = 0.440) had the highest standardized *β*-coefficients in combined multivariable regression models (0.447 and 0.440, respectively).

**Conclusions:**

IgE specificity, SA, avidity, and peanut diversity were greater in PA than in PS individuals. IgE peanut SA and peanut diversity had the greatest influence on effector cell activation and could be used clinically.

## Graphical abstract

**Figure:**
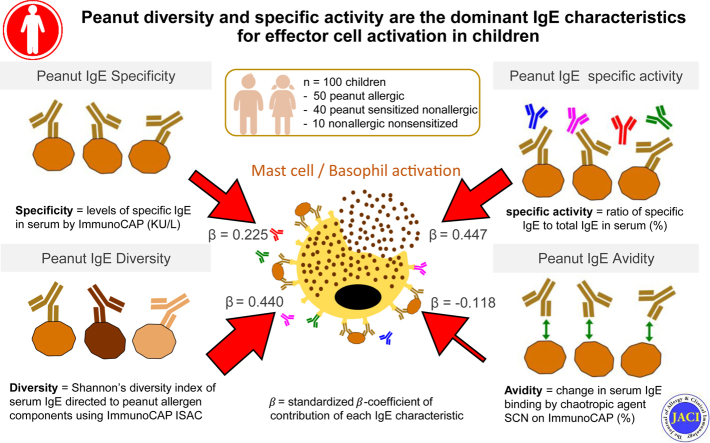

Allergen-specific IgE (sIgE) detection as a diagnostic tool reports many false-positives.^1^^,^^2^ For some foods, including peanut, sIgE testing has improved dramatically over recent years through incorporation of allergen components.^3^^,^^4^ Alongside component testing*, in vitro* effector cell assays, such as the basophil activation test (BAT), have shown better diagnostic accuracy and discrimination between allergic and sensitized but tolerant individuals.^5^ A possible explanation for the superiority of BAT in reflecting individuals’ food-allergic phenotype is that it considers all characteristics of sIgE, rather than only levels, as well as possible blocking antibodies and intrinsic basophil sensitivity.^6^^,^^7^ The mast cell activation test (MAT) is also more specific than sIgE alone^8^ and has the advantage of not requiring fresh blood like the BAT. Because the mast cells are homogeneous and propagated from a single human mast cell,^9^ sensitized with plasma from different subjects, and washed before allergen stimulation, the MAT is well suited as an *in vitro* model to study the functional characteristics of IgE.

Examples of intrinsic functional characteristics of IgE likely to influence effector cell degranulation in response to allergen are specificity, specific activity (SA), diversity, and affinity.^6^ IgE specificity is known to correlate with allergic status, illustrated by the dominance of Ara h 2 sIgE as a diagnostic marker in peanut allergy.^3^^,^^4^^,^^10^ Conversely, levels of total IgE (tIgE) alone are considered useless as standalone in the diagnosis of specific allergies; however, the proportion of sIgE to tIgE, known as IgE SA, which reflects the proportion of IgE bound to effector cells that is specific for a given allergen, may be more informative.^11^, ^12^, ^13^ An individual’s reactivity to allergen is likely affected by the proportion of non-sIgE and its occupation of high affinity IgE receptors (FcεRI) on the surface of effector cells.^11^^,^^12^ Perhaps, an increased diversity in IgE repertoire is likely to dampen an allergen-specific response through the reduced chance of crosslinking on the cell surface.^13^ Conversely, IgE diversity to different epitopes within 1 allergen has been shown to increase basophil sensitivity to allergen and can possibly increase basophil reactivity.^14^, ^15^, ^16^ Both IgE diversity and basophil reactivity have been associated with severity of allergic reactions to peanut. IgE affinity for allergen is likely to influence effective IgE crosslinking able to cause effector cell activation.^17^ For polyclonal IgE responses to multivalent allergens, avidity, which refers to the collective effect of a combination of antigen-antibody interactions, reflects more accurately the strength with which IgE binds to allergen than affinity, which refers to molecular interactions between IgE and its epitopes.^18^

In this study, we aimed to determine the relative importance of IgE characteristics through the assessment of their diagnostic accuracy, influence on basophil and mast cell degranulation, and interplay with each other.

## Methods

### Study population

Peanut-allergic (PA), peanut-sensitized but tolerant (PS), and nonsensitized nonallergic (NA) individuals were evaluated as previously described.^5^ Peanut allergy was determined by positive response to oral food challenge, except for patients who had convincing clinical history of systemic allergic reactions within 1 year of sample collection, together with a skin prick test (SPT) wheal size greater than or equal to 8 mm and/or peanut-sIgE greater than or equal to 15 KU_A_/L.^19^ Peanut tolerance was defined by a negative oral food challenge or the ability to ingest greater than or equal to 4 g of peanut protein, twice weekly, without demonstrating an allergic response, as assessed by a validated peanut consumption questionnaire.^20^ Peanut sensitization was determined by SPT wheal size greater than or equal to 1 mm and/or a peanut-sIgE greater than or equal to 0.1 kU_A_/L. Children who were clinically unwell, had significant chronic illness, or were unwilling to participate were not included. One hundred patients were selected consecutively in chronological order of recruitment based on availability of plasma samples with sufficient volume. The study was approved by the South East London Research Ethics Committee 2, and written informed consent was obtained from parents of all children.

### Allergen-sIgE measurements

ImmunoCAP ISAC (Immuno Solid-phase Allergen Chip) (Thermo Fisher, Uppsala, Sweden) was carried out according to manufacturer’s instructions. IgE binding to each allergen array was recorded using a LuxScan 10K scanner (Core Life Sciences, Irvine, Calif) and data analyzed using the Phadia Microarray Image Analysis Software (Thermo Fisher, Uppsala, Sweden).

### IgE SA determination

IgE SA was determined by calculating the ratio of allergen-sIgE to tIgE levels, measured using the ImmunoCAP assays as detailed above, and expressed as percentage, using the following formula:

$$\frac{\text{sIgE to peanut}/\text{Ara h }1/2/3/6/8/9}{\text{tIgE}}\times 100$$

### Avidity measurements

**Fig E1:**
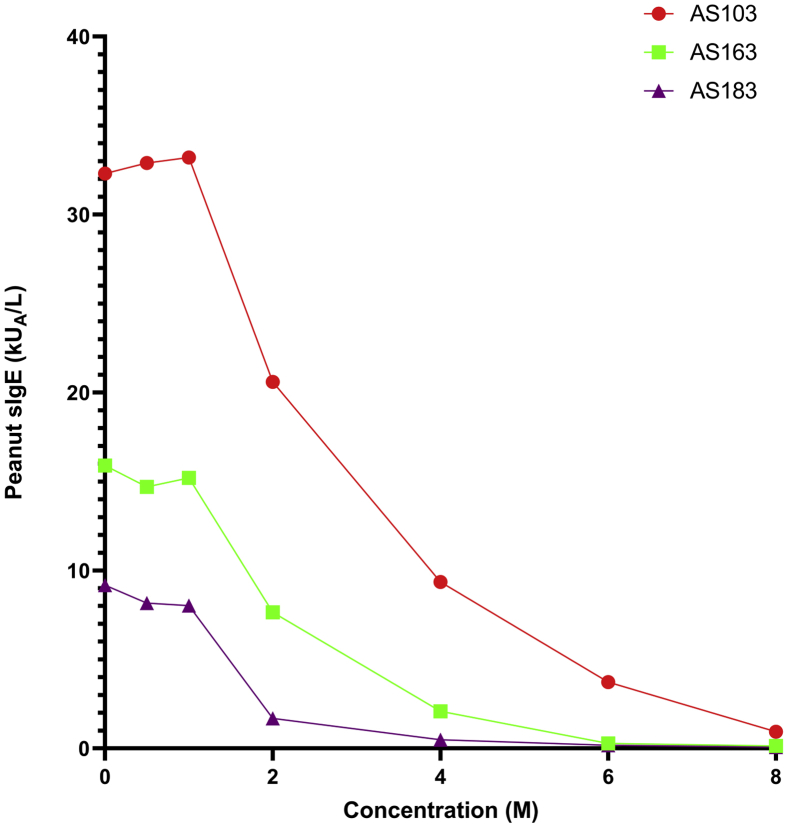
Avidity ImmunoCAP development assay incorporating 2 PA individuals and 1 PS individual. Titration of sodium thiocyanate (NaSCN) using the following concentrations: 0 molar (PBS), 0.5 molar, 1 molar, 2 molar, 4 molar, 6 molar, and 8 molar.

Avidity assays were performed to estimate the cumulative affinities of peanut-sIgE in plasma of both PA (n = 36) and PS (n = 24) patients (see Table E1 in this article's Online Repository at www.jacionline.org). The assay was developed on the basis of avidity ELISAs using thiocyanate solutions to interfere with antibody:antigen interactions.^21^^,^^22^ To determine the optimal concentration of sodium thiocyanate (NaSCN), plasma samples from 3 individuals, 1 PA individual (AS103) with peanut-sIgE levels closer to top of detection limit (100 kUA/L), 1 PA individual (AS163) with peanut-sIgE levels toward the lower detection limit of the assay, however with enough IgE to allow accurate inhibition (∼20 kUA/L), and 1 PS individual (AS183) with similar peanut IgE levels (∼20 kUA/L). We assayed plasma from each individual, in a 1:1 ratio, with NaSCN from 0 to 8 molar concentrations. Two molar NaSCN was selected as the optimal concentration, as it was the lowest concentration to cause measurable reduction in IgE binding, across both PA and PS individuals (see Fig E1 in this article’s Online Repository at www.jacionline.org). For avidity assays across the whole cohort, samples were selected on the basis of volume and peanut-sIgE level greater than or equal to 1 kU_A_/L. This minimum detection limit (≥1 kU_A_/L) was based on linearity experiments, in which serial dilutions spanning the detection limit of the whole assay were measured. For avidity estimations, patient plasma was incubated for 20 minutes at room temperature, with either PBS (negative control) or 2 molar NaSCN. Preincubated samples were then assayed for peanut-sIgE using ImmunoCAP. Avidity results were reported as “100 − %binding decrease” following NaSCN incubation and calculated using the formula below:

$$100-\frac{\left(\text{sIgE level w}/\text{NaSCN}\right)}{\text{sIgE level w}/\text{PBS}}\times 100$$

### Diversity measurements

The diversity of an individual’s IgE repertoire was generated from ImmunoCAP ISAC assays. Shannon’s diversity index^23^ was used to transform the data as follows:

$$H=-\sum_{i=1}^{S}P_{i}\;\ln\;P_{i}$$

where *H* is the diversity index and is the reported output; *i* = (1, ... *S*), *S* is the total number of allergens measured, and *P*_*i*_ is the proportion (relative abundance) of allergen, *i*, in total amount of allergen-sIgE levels.

### Basophil and mast cell activation assays

Whole-blood BAT and MAT were performed as previously described,^5^^,^^8^^,^^24^ with BAT performed on 54 individuals (PA = 23, PS = 31) and MAT performed on 100 individuals (PA = 50, PS = 40, NA = 10). For the BAT, heparinized whole blood was stimulated for 30 minutes at 37°C with peanut extract diluted in RPMI media, resulting in serial 10-fold dilutions from 10 μg/mL to 0.1 ng/mL. Anti-IgE (1 μg/mL) and N-Formylmethionine-leucyl-phenylalanine (fMLP, 1 μM) were used as positive controls and RPMI alone as the negative control. For the MAT, Laboratory of Allergic Diseases 2 (LAD2) cells^9^ were cultured in rIL-4 5 days before overnight sensitization with patient plasma, as previously described. Serial dilutions of peanut extract (0.1-1000 ng/mL) or controls (anti-IgE 1 μg/mL, ionomycin 1 μg/mL, or 0.04% BSA RPMI) were added to presensitized cells and incubated for 1 hour at 37˚C. Basophils were identified as SSClow/CD203c^+^/CD123^+^/HLA-DR^−^ and mast cells as forward scatter/viable cells. For both BAT and MAT, surface expression of CD63 was measured by flow cytometry using FACS Canto II with FACSDiva software (BD Biosciences, San Diego, Calif) and data were analyzed using FlowJo software version 7.6.1 (TreeStar, Ashland, Ore).

### Statistical analyses

Comparisons of groups were done using Mann-Whitney *U* test and Kruskal-Wallis *P* tests. The diagnostic performance of all measurements was examined against allergic status to peanut using receiver-operating characteristic (ROC)-curve analyses. Associations between individuals’ characteristics and mast cell activation were analyzed using Spearman rank correlation, with mast cell activation distributed in a non-normal fashion. Linear regression models were generated for each characteristic of IgE investigated in this study, with their regression (*β*) coefficients from univariable analyses reported in Table II. Multivariable linear regression models were designed including peanut-sIgE levels, peanut SA, peanut avidity, peanut diversity (PD), and whole diversity (WD), in a number of combinations as outlined in Table III. For standardized beta (*β*) coefficients, input data were standardized by generating *Z* scores, using the following formula, before performing linear regression analyses:

$$Z=\frac{x-\mu }{\sigma }$$

where *Z* is standardized data point, *x* is observed data point, *μ* is mean of sample, and *σ* is SD of the sample. Statistical analyses were performed using GraphPad Prism 7.0 (GraphPad Software, San Diego, Calif), SPSS 25.0 (IBM SPSS Statistics, New York, NY), and MedCalC 19.4.1 (MedCalc Software, Ostend, Belgium).

## Results

### Study population

One hundred children (50 PA, 40 PS, 10 NA), aged 5 months to 17 years, 69% male, were studied. PA individuals were significantly older than PS individuals (*P* = .003). All demographic, clinical, and serological features of the study population are presented in Table I.

**Table I:** Demographic and clinical features and serum sIgE levels of the study population (n = 100)

Demographic, clinical, and immunologic features	PA (n = 50)	PS (n = 40)	NA (n = 10)	*P* value
Age (y)	8.31 (1.68-17.91)	5.93 (0.52-15.81)	5.96 (0.77-12.58)	.003
Sex: male	36 (72)	25 (62.5)	8 (80)	.371
Atopic eczema	17 (34)	18 (45)	2 (20)	.287
Asthma/wheezing	29 (58)	13 (33)	1 (10)	.016
Allergic rhinitis	33 (66)	14 (35)	1 (10)	.003
Other food allergy	38 (76)	33 (83)	2 (20)	.453
SPT wheal size to peanut (mm)	9 (1-34)†	3 (0-12)†	0 (0-0)	<.0001
tIgE (kU_A_/L)	297.5 (4-3,550)	469 (7-10,714)	29.5 (6-397)	.5757
sIgE to peanut (kU_A_/L)	12 (0.2-568)	2.41 (0.04-128)	0.01 (0-0.9)	.0019
sIgE to Ara h 1 (kU_A_/L)	0.80 (0-199)	0.11 (0-88.3)	0 (0-0.01)	.0838
sIgE to Ara h 2 (kU_A_/L)	2.54 (0.01-278)	0.07 (0-82.3)	0.04 (0.01-0.07)	<.0001
sIgE to Ara h 3 (kU_A_/L)	0.36 (0-89.6)	0.06 (0-7.28)	0.01 (0-0.04)	.0456
sIgE to Ara h 6 (kU_A_/L)	3.835 (0-155)	0.03 (0-86)	0 (0-0.29)	<.0001
sIgE to Ara h 8 (kU_A_/L)	0.09 (0-185)	0.04 (0-88.6)	0 (0-0.02)	.4022
sIgE to Ara h 9 (kU_A_/L)	0.02 (0-871)	0.05 (0-43.9)	0.01 (0-0.02)	.0834
Peanut SA (%)	5.03 (0.06-81.71)	0.82 (0.02-7.68)	0.03 (0-0.18)	<.0001
Ara h 1 SA (%)	0.37 (0-24.34)	0.03 (0-2.12)	0 (0-0.04)	.0038
Ara h 2 SA (%)	2.48 (0.01-55.26)	0.012 (0-1.86)	0.09 (0.01-0.83)	<.0001
Ara h 3 SA (%)	0.14 (0-9.98)	0.019 (0-0.90)	0.01 (0-0.67)	.0002
Ara h 6 SA (%)	1.91 (0-25.63)	0.01 (0-1.06)	0 (0-0.41)	<.0001
Ara h 8 SA (%)	0.05 (0-11.90)	0.01 (0-7.77)	0 (0-0.04)	.2862
Ara h 9 SA (%)	0.01 (0-1.5)	0.01 (0-4.72)	0.01 (0-0.17)	.3529
PD	0.97 (0-1.41)	0 (0-1.35)	0 (0-0)	<.0001
WD	1.985 (0-3.39)	1.82 (0-3.1)	0 (0-1.32)	.4945
Avidity (%inhibition)‡	41.07 (15.94-72.5)	28.63 (7.43-50)	—	<.0001
MAT to peanut (%CD63^+^ LAD2 cells)	24.6 (0-79.9)	4.18 (0-31.65)	1.09 (0-3.9)	<.0001
BAT to peanut† (%CD63^+^ basophils)∗	37.55 (1.75-90.79)	1.57 (0-16.6)	0.36 (0-0.99)	<.0001

Values are expressed as n (%) or median (range). *P* value refers to the comparison between PA and PS patients.

∗ BAT = PA (n = 23), PS (n = 54), NA (n = 7).

† SPT = PA (n = 49), PS (n = 38), PA + PS (n = 87).

‡ For avidity analyses, n = 60, with PA (n = 36) and PS (n = 24).

### Peanut-sIgE is strongly correlated with mast cell activation

**Fig E2:**
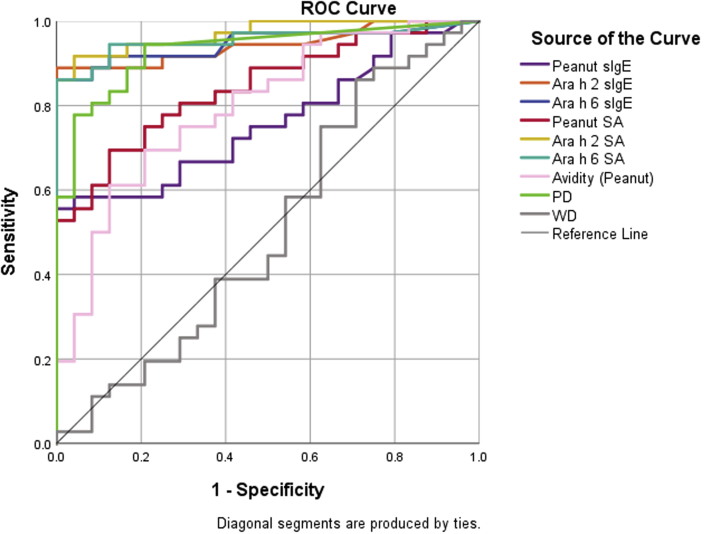
ROC curves for the most discriminative characteristics, allowing for comparison, in the subpopulation of n = 60, initially selected for avidity analyses. Displayed are ROC curves for peanut-sIgE (purple), Ara h 2 sIgE (orange), Ara h 6 sIgE (blue), peanut SA (red), Ara h 2 SA (yellow), Ara h 6 SA (turquoise), avidity (pink), PD (light green), and WD (gray). For corresponding areas under the curves, see Table E4.

We previously reported that sIgE levels to peanut (*P* < .01), Ara h 2 (*P* < .0001), Ara h 3 (*P* = .046), and Ara h 6 (*P* < .0001) were significantly higher in PA than in PS individuals, with Ara h 2 and Ara h 6 showing the greatest diagnostic utility.^10^ The other components tested did not discriminate between PA and PS individuals (see Fig E2 in this article’s Online Repository at www.jacionline.org).

**Fig E3:**
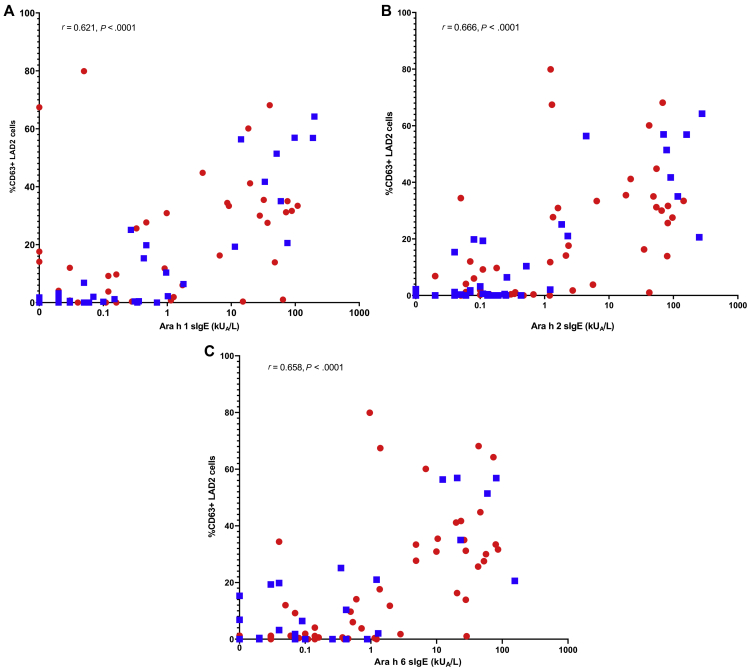
Spearman correlation between (**A**) Ara h 1, (**B**) Ara h 2, (**C**) Ara h 6 sIgE levels and mast cell activation. %CD63^+^ LAD 2 cells represent cell activation following the MAT. Correlation reported across peanut-sensitized population (n = 90), with PA individuals (red) and PS individuals (blue). *R* values have been displayed on the figure and in Table II.

Here, we assessed to what degree the levels of sIgE correlated with peanut-induced mast cell activation. Peanut-sIgE was strongly correlated with peanut-induced mast cell activation, reporting an *R* value of 0.787 (*P* < .0001), with Ara h 2 reporting 0.666 (*P* < .0001), Ara h 6 0.658 (*P* < .0001), and Ara h 1 0.621 (*P* < .0001) (Fig 3, *A*; see Fig E3 in this article’s Online Repository at www.jacionline.org; Table II). A full characteristic correlation matrix, with corresponding *P* values, has been reported in Tables E2 and E3 in this article’s Online Repository at www.jacionline.org.

**Table II:** Summary table reporting the discriminative ability and association of each characteristic with mast cell activation (%CD63^+^ LAD2 cells)

Characteristic	PA vs PS *t* test *P* value (*P* value adjusted for multiple comparisons∗)	Correlation						Linear regression (n = 90)				
		*R* value (n = 90)	*P* value (n = 90)	*R* value (PA, n = 50)	*P* value (PA, n = 50)	*R* value (PS, n = 40)	*P* value (PS, n = 40)	*Β*-coefficient	Coefficient SE	Standardized *β*-coefficient	*P* value	*R*^2^ value
Peanut-sIgE (kU_A_/L)	.0019 (.030)	0.787	<.0001	0.716	<.0001	0.702	<.0001	0.094	0.016	0.521	<.0001	0.271
Ara h 1 sIgE (kU_A_/L)	.0838 (1)	0.621	<.0001	0.637	<.0001	0.475	.0019	0.307	0.050	0.551	<.0001	0.304
Ara h 2 sIgE (kU_A_/L)	<.0001 (<.0001)	0.666	<.0001	0.641	<.0001	0.208	.1977	0.212	0.037	0.526	<.0001	0.268
Ara h 3 sIgE (kU_A_/L)	.0456 (.7296)	0.613	<.0001	0.587	<.0001	0.556	.0002	0.490	0.118	0.405	<.0001	0.164
Ara h 6 sIgE (kU_A_/L)	<.0001 (<.0001)	0.658	<.0001	0.625	<.0001	0.205	.2042	0.384	0.074	0.484	<.0001	0.234
Ara h 8 sIgE (kU_A_/L)	.4022 (1)	0.385	.0001	0.311	.0280	0.444	.0041	−0.065	0.091	−0.078	.463	0.006
Ara h 9 sIgE (kU_A_/L)	.0834 (1)	0.219	.0385	0.237	.0975	0.445	.0036	0.146	0.410	0.038	.722	0.001
Peanut SA	<.0001 (<.0001)	0.659	<.0001	0.604	<.0001	0.485	.0015	0.980	0.129	0.628	<.0001	0.395
Ara h 1 SA	.0038 (.0608)	0.502	<.0001	0.580	<.0001	0.158	.3316	2.722	0.361	0.626	<.0001	0.386
Ara h 2 SA	<.0001 (<.0001)	0.515	<.0001	0.523	.0001	−0.209	.1950	1.494	0.245	0.545	<.0001	0.297
Ara h 3 SA	.0002 (.0032)	0.418	<.0001	0.457	.0008	0.024	.8847	4.231	1.224	0.346	<.0001	0.120
Ara h 6 SA	<.0001 (.0001)	0.513	<.0001	0.411	.0030	−0.031	.8533	2.194	0.455	0.455	<.0001	0.209
Ara h 8 SA	.2862 (1)	0.146	.169	0.012	.2957	0.194	.2302	0.045	1.055	0.005	.966	0.000
Ara h 9 SA	.3529 (1)	−0.008	.942	−0.081	.6262	0.248	.1219	−2.850	3.569	−0.085	.427	0.007
PD	<.0001 (<.0001)	0.649	<.0001	0.565	<.0001	0.233	.1477	23.763	3.085	0.635	<.0001	0.396
WD	.4945 (1)	0.494	<.0001	0.442	.0006	0.545	.0002	8.362	2.525	0.333	.001	0.111
Avidity (%inhibition)†	<.0001	0.236	.069	−0.207	.1168	−0.344	.0999	0.267	0.177	0.174	.183	0.036

Discriminative ability was assessed using Mann-Whitney *t* test and associations with mast cell activation are reported as both Spearman rank (*R* value) correlation and logistic regression (*β*-coefficient). *P* value refers to the comparison between PA and PS patients.

∗ For avidity analyses n = 60, with PA (n = 36) and PS (n = 24).

† Adjustment for multiple comparisons was done with Bonferroni-Dunn.

### IgE SA improves the discrimination between PA and PS

**Fig 1:**
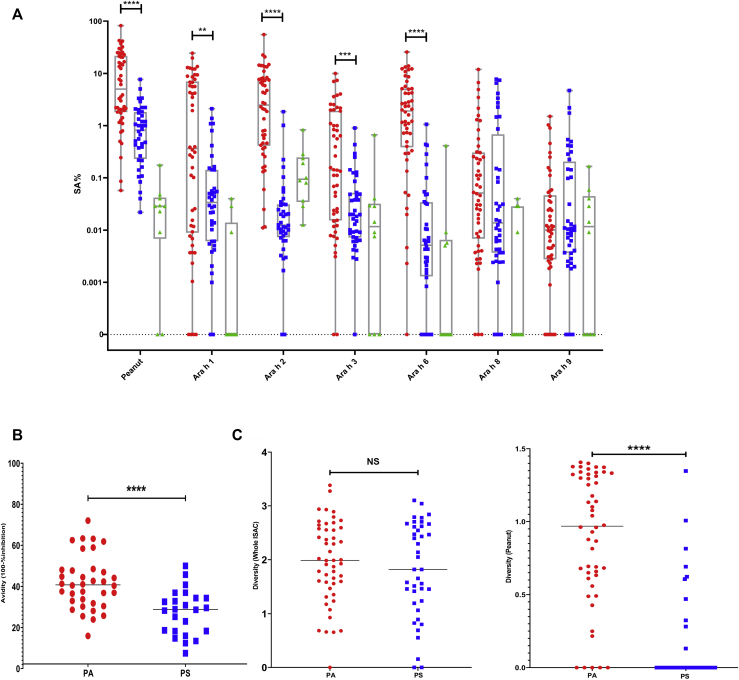
Distribution of IgE (**A**) SA, (**B**) avidity for peanut, and (**C**) diversity in PA individuals (in red, n = 50), PS individuals (in blue, n = 40), and NA individuals (in green, n = 10). The Mann-Whitney *U* test was used for the comparison between PA individuals and PS individuals. *NS*, Not significant; *ISAC*, Immuno Solid-phase Allergen Chip. ∗*P* < .05; ∗∗*P* < .01; ∗∗∗*P* < .001; ∗∗∗∗*P* < .0001.

**Fig 2:**
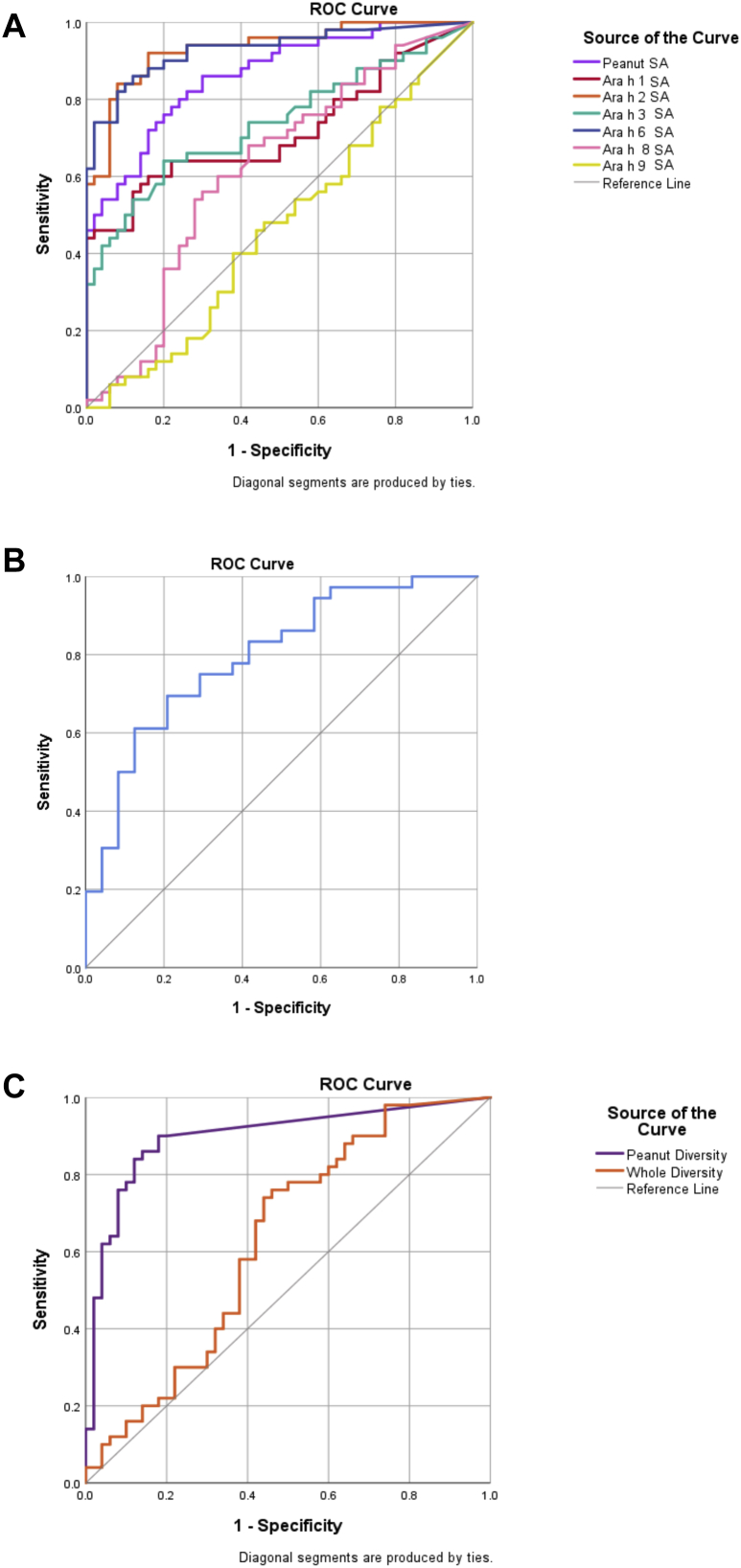
ROC curves for IgE (**A**) SA, (**B**) peanut avidity, and (**C**) diversity across the whole study population (n = 100). For corresponding AUCs, see Table II. Labels have been included in the figure, where necessary.

**Fig 3:**
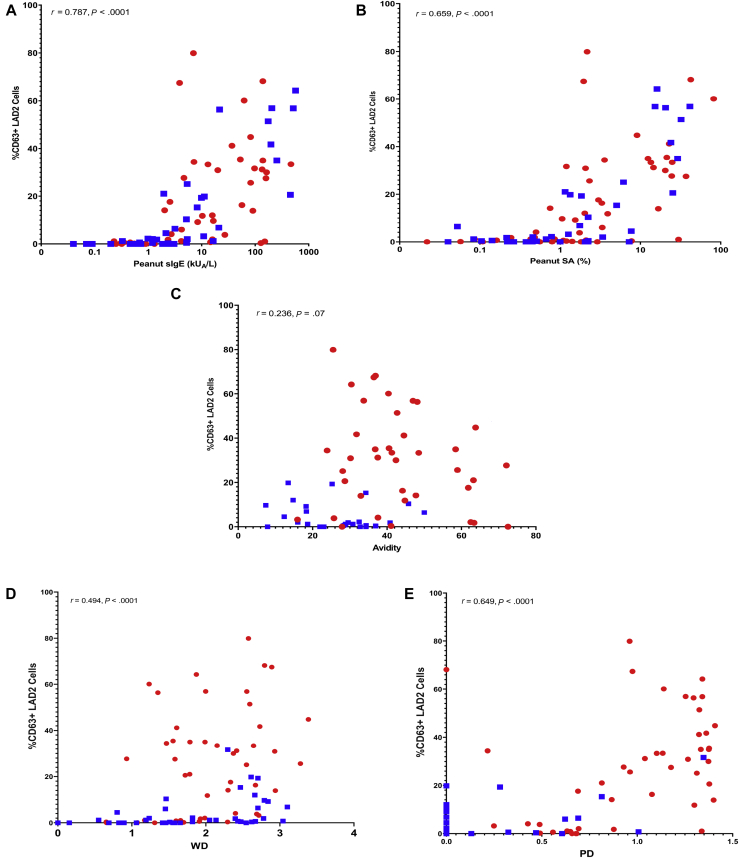
Spearman’s correlation between (**A**) peanut-sIgE levels, (**B**) peanut SA, (**C**) peanut avidity, (**D**) WD, and (**E**) PD, and mast cell activation. %CD63^+^ LAD 2 cells represent cell activation following the MAT. Correlation reported across peanut-sensitized population (n = 90), with PA individuals (red) and PS individuals (blue). *R* values have been displayed on the figure and in Table II.

IgE SA values to peanut (*P* < .0001), Ara h 1 (*P* = .0038), Ara h 2 (*P* < .0001), Ara h 3 (*P* = .02), and Ara h 6 (*P* < .0001) were higher in PA than in PS individuals (Fig 1, *A*; Table II). In ROC analyses, peanut and allergen components showed a larger area under the ROC curve for SA than for sIgE alone—for instance, Ara h 2 SA had an area under the curve (AUC) of 0.933, followed by Ara h 6 (0.930), whole peanut (0.862), Ara h 3 (0.738), and Ara h 1 (0.716), (Fig 2, *A*; see Table E4 in this article’s Online Repository at www.jacionline.org), with peanut SA showing a significantly higher AUC (*P* = .0038) than peanut-sIgE, in the same population (see Table E5 in this article’s Online Repository at www.jacionline.org). SA correlated with mast cell activation across both PA and PS individuals (n = 90), with peanut SA reporting the highest *R* value of 0.659 (*P* < .0001), followed by Ara h 2 (0.515, *P* < .0001) and Ara h 6 (0.513, *P* < .0001) (Fig 3, *B*; see Fig E3 in this article’s Online Repository at www.jacionline.org; Table II).

### Avidity of IgE for peanut is higher in patients with peanut allergy and correlates with mast cell activation

PA individuals (n = 36) showed a significantly higher avidity score (mean ± SD, 41.82 ± 13.00 %inhibition of IgE binding) than PS (n = 24) individuals (mean ± SD, 27.41 ± 10.95) (*P* < .0001, Mann-Whitney *U* test); however, there was an overlap between the groups (Fig 1, *B*). The avidity ROC analyses exhibited an AUC of 0.795 (Fig 2, *B*; see Tables E4 and E6 in this article’s Online Repository at www.jacionline.org). There was a very weak, but insignificant, correlation between the avidity of peanut-sIgE and mast cell activation in response to peanut (*R* = 0.236; *P* = .069; Fig 3, *C*).

### IgE diversity for peanut allergens strongly correlates with mast cell activation

To measure IgE diversity, we transformed the ISAC data using Shannon index to create 2 variables: WD, incorporating all 112 allergens, and PD, considering the peanut allergens Ara h 1, 2, 3, 6, 8, and 9. Diversity within the peanut allergens was significantly greater (*P* < .0001) in PA (mean, 0.89) than in PS (mean, 0.16) individuals; however, WD did not discriminate (*P* = .495) between PA (mean, 1.96) and PS (mean, 1.81) individuals (Fig 1, *C* and *D*). ROC analyses were performed to see how these variables were distinct in allergic versus nonallergic subjects (Fig 2, *C*; Table E4). The AUC was 0.898 for PD and 0.628 for WD. PD also reported greater correlation with mast cell activation (*R* = 0.649; *P* < .0001) compared with WD (*R* = 0.494; *P* < .0001) (Fig 3, *D* and *E*).

### Peanut IgE diversity and SA are the major determinants of mast cell and basophil activation

Having studied each characteristic of IgE separately, we assessed their relative importance and influence on effector cell activation. We generated logistic regression models incorporating the most important IgE characteristics based on their ability to discriminate between the PA and PS populations, their correlation with LAD2 cell activation, and significant (*P* < .2) standardized *β*-coefficients in simple logistic regression (Table III).

**Table III:** Summary of mechanistic multivariable linear regression models (n = 60), in which combinations of functional IgE characteristics were used to predict %CD63^+^ LAD2 cell activation

Model no.	Characteristic	Unstandardized *β*- coefficient	Coefficient SE	Standardized *β*-coefficient	*P* value	*R*^2^ value	VIF
1	Peanut-sIgE	0.037	0.020	0.225	.066	0.415	1.384
	PD	20.528	5.203	0.534	<.0001		1.750
	Avidity	−0.141	0.180	−0.092	.436		1.322
2	Peanut SA	0.664	0.156	0.447	<.0001	0.530	1.314
	PD	16.940	4.470	0.440	<.0001		1.608
	Avidity	−0.181	0.158	−0.118	.258		1.275
3	Peanut-sIgE	0.077	0.019	0.472	<.0001	0.255	1.008
	WD	2.171	4.306	0.057	.628		1.035
	Avidity	0.223	0.180	0.145	.224		1.043
4	Peanut SA	0.993	0.154	0.668	<.0001	0.447	1.084
	WD	7.383	3.786	0.201	.056		1.078
	Avidity	0.121	0.157	0.079	.444		1.063

Unstandardized and standardized *β*-coefficients are reported alongside significance and variance inflation factor (VIF). VIF >10 was used to indicate any collinearity.

Four multivariable mechanistic logistic regression models were designed, incorporating 3 characteristics dissimilar in nature and acquisition, to reduce the probability of collinearity. Model 1 incorporated peanut-sIgE, PD, and avidity, generating standardized *β*-coefficients of 0.225 (*P* = .066), 0.534 (*P* < .0001), and −0.092 (*P* = .436), respectively. This suggested peanut IgE diversity to peanut allergens was the most influential characteristic of IgE in mast cell activation. This effect was mirrored when assessing basophil activation (see supplementary model s5), albeit with a greater relative influence reported by PD. In model 2, peanut-sIgE was replaced with peanut SA, and the influence that PD had on effector cell activation was lower compared with model 1 but still statistically significant. Peanut SA, PD, and avidity displayed coefficients of 0.447 (*P* < .0001), 0.440 (*P* < .0001), and 0.118 (*P* = .2580), respectively, with both PD and peanut SA reporting a significant impact on effector cell activation.

Models 3 and 4 mirrored models 1 and 2; however, WD was incorporated to replace PD. In model 3, peanut-sIgE was the only characteristic to influence effector cell activation with any significance, exhibiting a standardized *β*-coefficient of 0.472 (*P* < .0001). A similar effect was seen in model 4, with peanut SA replacing peanut-sIgE. Similar trends were seen considering basophil activation in models S6 and S7. Peanut SA influenced activation to a greater extent, reporting a standardized *β*-coefficient of 0.668 (*P* < .0001). Models 3 and 4 showed the positive impact peanut avidity measurements may have in the prediction of effector cell activation, albeit with poor significance. Supplementary models were designed to directly compare the influence of similar characteristics (see Table E7 in this article’s Online Repository at www.jacionline.org). Of note, supplementary model S2, containing only peanut-sIgE and SA, reports SA with a greater standardized *β*-coefficient and significance.

Given that the biology of primary basophils can differ from LAD2 cells, namely in terms of IgE receptor density and intrinsic cellular sensitivity, which can vary between patients, we assessed the relative importance of the same IgE characteristics on basophil activation. Univariable and multivariable logistic regression models were fitted using the available whole-blood BAT results in the same patient population (n = 54) (Table I).^5^ Both peanut SA and PD reported the highest standardized *β*-coefficients across both univariable, 0.762 (*P* < .0001) and 0.843 (*P* < .0001), respectively, and multiple multivariable regression models, such as supplementary model 4, reporting standardized *β*-coefficients of 0.335 and 0.690, respectively (see Tables E8 and E9 in this article’s Online Repository at www.jacionline.org).

## Discussion

Levels of allergen-sIgE do not always reflect clinical reactivity to peanut, which raises the hypothesis that, beyond levels, the qualitative characteristics of sIgE may influence effector cell response to peanut allergens. In this study, we tested samples from a well-characterized cohort of 100 children being assessed for peanut allergy on the BAT and MAT to peanut, which reflect *in vitro* the clinical reactivity to peanut,^8^ and quantified SA, diversity, and avidity of IgE for peanut allergens in each subject. PA children had higher proportion of allergen-sIgE, higher diversity, and higher avidity of IgE for peanut allergens. These variables, as well as peanut and peanut component sIgE, correlated with effector cell activation to a greater or lesser extent. Using multivariable logistic regression models, the IgE characteristics that appeared to be most determinant of effector cell activation following peanut stimulation were SA and PD, suggesting that the IgE repertoire bound to receptors on the surface of mast cells is key in influencing degranulation. The greater proportion of allergen-sIgE and the broader array of peanut allergens that IgE recognizes, the greater the mast cell activation in response to peanut and, consequently, the greater the likelihood and severity of peanut allergy.^8^ To our knowledge, this is the first study assessing the functional characteristics of polyclonal-sIgE in a patient population, as well as the first study to quantify avidity using ImmunoCAP and to introduce the concept of diversity as a quantitative variable, which showed unprecedented influence on effector cell activation.

Quantifying allergen-sIgE has shown diagnostic utility in that elevated levels are associated with a higher likelihood of food allergy.^25^ Component-sIgE and epitope-sIgE detection added further resolution in peanut allergy diagnosis.^3^^,^^4^^,^^26^, ^27^, ^28^ We recently showed the importance of IgE specificity to peanut 2S albumins, Ara h 2 and Ara h 6, with Ara h 2 being the most dominant IgE-binding allergen and inducer of effector cell activation.^10^ Unsurprisingly, in this study, we made similar observations. However, because whole peanut extract was used as the stimulant in the MAT and whole peanut extract represents better the peanut ingested by patients, peanut-sIgE levels, rather than sIgE to peanut components, were used to represent specificity across multivariable models. The peanut components included Ara h 8 and also Ara h 9, which are less likely to cause systemic reactions, but we opted to include them in the analyses, because, from a mechanistic perspective, low-affinity interactions can contribute to effector cell degranulation.^29^

We used 2 *in vitro* systems to test the functionality of IgE and use as end points for the univariate and multivariate models: the BAT and the MAT. The use of the 2 cell types is an important validation of our findings. We used the LAD2 cells as the mast cells in the MAT.^8^ The uniformity of using human mast cell line removes the heterogeneity that others have seen when using primary mast cells,^30^ and allows us an excellent functional effector cell platform for exploring isolated IgE characteristics, with the least possible “noise” from mast cell–intrinsic features.^30^ Moreover, the MAT assay isolates receptor-bound antibodies, through initial wash steps, removing unbound antibodies from assay. This ablates the effect of blocking antibodies in the periphery, such as allergen-specific IgG_4_, and encourages the assessment of receptor-bound IgE.^24^

We report that peanut-sIgE significantly influences both basophil and mast cell activation, as exemplified in the univariable logistic regression models and multivariable models 1, 3, and S6. Impressively, SA improved discrimination between PA and PS individuals and had greater influence on both basophil and mast cell activation. It can be observed for some components, such as Ara h 2, that SA in the NA group reports higher medians than the PS group. This is due to the difference in tIgE levels between the 2 groups, in which tIgE is typically within the normal range in the NA group, and largely elevated in the PS group due to their atopic nature. The importance of SA has been highlighted by Christensen et al,^6^ showing that higher relative concentrations of allergen-sIgE to nonallergen sIgE increased both basophil sensitivity and maximum basophil degranulation. Clinically, IgE SA has been shown as a strong predictor in the outcome of oral food challenges^11^ and the efficacy of anti-IgE treatment in allergic individuals.^31^ However, other studies have failed to demonstrate improved diagnostic performance and showed comparable AUCs for both sIgE levels and SA to peanut, and peanut components.^32^ In our study, we report a significant increase in AUCs with peanut SA compared with standalone peanut-sIgE, suggesting that determining sIgE:tIgE ratios may have diagnostic benefits. Moreover, the increased influence on effector cell activation shown by incorporating peanut SA, over peanut-sIgE, in multivariable regression models also supports the utility of SA as a better predictor of allergic reactivity. The direct comparison of peanut-sIgE and SA in single models, S2 and S9 (Tables E5 and E6), further substantiates SA’s dominance as an influential characteristic over standalone sIgE titers. Conceptually, an increase in IgE SA or ratio of relevant sIgE to irrelevant sIgE acts to increase the probability that 2 IgE antibodies attached to FcεRI will become complexed with an allergenic molecule, thus inducing degranulation.

It is well established that high-affinity binding of IgE to allergen is a characteristic that influences the mechanisms of allergic reactions, and has been demonstrated across both experimental and clinical studies.^17^^,^^33^ It has recently been reported that production of high-affinity IgE is regulated by a distinct subset of previously unidentified T follicular helper cells, characterized by their high IL-13 expression.^34^ High-affinity allergen-sIgE is required to outcompete lower affinity allergen-specific-IgG and initiate the allergic response.^35^ Despite this, a role for low-affinity interactions has been previously demonstrated using both high- and low-affinity IgE of same specificity and showing marked reduction in effector cell degranulation in response to inhibition of only low-affinity sIgE.^31^^,^^36^ This implies that summative avidity of allergen-sIgE is a potentially more important consideration than the affinity of individual IgE molecules.

The development of avidity ELISAs has enabled the estimation of avidity in polyclonal samples, by using different chaotropic agents.^21^^,^^37^, ^38^, ^39^, ^40^ We applied these principles using the well-established, validated, and fully quantitative ImmunoCAP technology. We showed a high degree of significant discrimination between PA and PS individuals, in which peanut-sIgE from PA individuals was far more tolerant of NaSCN addition than peanut-sIgE from PS individuals, with a greater proportion remaining bound to peanut ImmunoCAP. A recently published study suggested that this difference in avidity between clinically distinct sensitized populations could be a consequence of allergen exposure, with chronic allergen exposure leading to the accumulation of long-lived plasma cells, generating higher affinity IgE.^41^ We expected that our data for peanut-sIgE avidity would have higher impact on effector cell activation. This may be a consequence of limitations within the variable itself. We proposed a fully quantitative method for determining IgE avidity in patient samples, in which removal of low-avidity interactions in the presence of NaSCN was not influenced by the levels of sIgE in the sample. However, unlike the other variables tested, the avidity score is presented as relative inhibition, and therefore relies heavily on the original peanut-sIgE level, determined in the absence of NaSCN. We believe that IgE avidity can become more influential in cases in which IgE levels/concentrations and diversity are comparable between allergic and nonallergic individuals.

The diversity of one’s IgE repertoire as an influential characteristic in the mechanisms of allergy is lesser established. Allergen-sIgE levels, from both the whole allergen and the components perspectives, are exceptionally informative for the diagnosis and mechanistic understanding of peanut allergy; however, we do not normally consider the specificity of remaining IgE. Over recent years, the phenomena of “epitope/molecular spreading” has been described in allergy, whereby IgE specificity and concentration, to components/epitopes within clinically relevant allergens, diversifies over time and creates a more heterogeneous pool of allergen-sIgE.^14^^,^^42^^,^^43^ We used ImmunoCAP ISAC technology and designed variables, based on Shannon diversity, to reflect the diversity and concentration of IgE to both a wide array of allergens (WD) and allergen components within peanut (PD).^44^^,^^45^ When applying Shannon’s diversity principles to our population, WD did not discriminate between PA and PS individuals. This was not an unexpected finding, because most children in our cohort were highly atopic presenting with multiple allergic comorbidities. However, because both PA and PS individuals had detectable IgE to peanut and peanut major allergens, we could have expected a similar outcome with PD, but this was not the case because PD was able to discriminate between these cohorts in a highly significant manner. Moreover, PD showed unusually high diagnostic value and correlated well with mast cell activation. Perhaps most impressive was the influence that PD had on effector cell activation, where in simple logistic regression models, PD reported the outright highest standardized *β*-coefficients (*P* < .0001), reflecting influence on both mast cell and basophil activation. In multivariable mechanistic models, PD and peanut SA outperformed avidity in model 2, reporting comparably significant influence on mast cell activation, but with PD reporting slightly more significant impact on basophil activation. When plotted in isolation with peanut SA, PD reported a slightly greater standardized *β*-coefficient, considering both mast cell and basophil activation. The molecular spreading phenomenon is the most viable rationale for higher PD, in PA compared with PS individuals. This phenomenon has been described in children with hay fever, whereby *Phleum pratense* sensitization across different allergenic molecules increases/diversifies temporally, following the initial onset of disease.^43^ Under the assumption that some PS children eventually develop clinical peanut allergy, we could predict an increase in their IgE diversity to peanut allergens over time with progression of their allergy. Similar observations have been reported in the Learning Early About Peanut allergy (LEAP) study.^46^

Overall, for each of the IgE characteristics measured, there was a significant difference between PA and PS individuals, all showing improved diagnostic utility over peanut-sIgE levels and influenced effector cell activation. Between many of these variables, significant correlation was reported, reflecting the fact that allergic individuals tend to report high levels/scores across different functional IgE characteristics, namely higher levels of peanut-sIgE, higher peanut SA, higher PD, and higher avidity for peanut allergens. Similarly, as discussed, many PA individuals also report polysensitization to the different peanut allergens. This can often introduce concerns of multicollinearity when building multivariable models. In our study, IgE characteristics were included in the same model only if dissimilar in nature and acquisition, with collinearity presumed minimal as demonstrated by the low variance inflation factor scores. Our conclusions require, nevertheless, verification in future studies. In our cohort of 90 subjects with detectable peanut-sIgE, PD and peanut SA held the most influence on effector cell activation, with SA offering improved diagnostic accuracy over peanut-sIgE levels. It is likely that mast cell reactivity, and thus clinical peanut allergy, is a consequence of multiple IgE characteristics and none should be discounted in further investigations into the mechanisms of food allergy. Other IgE characteristics, such as glycosylation/posttranslational modifications, should be investigated, with the inhibitory effect of desialyation of glycans on IgE from allergic individuals potentially leading to therapeutic benefits.^47^ The clinical implications of IgE characteristics may extend to the effect of immunomodulatory treatments, such as allergen-specific immunotherapy^48^ and biologics, and could be used as biomarkers of response to treatment.

**Key messages**

• IgE specificity, SA, avidity, and diversity are significantly different between PA and PS individuals and are positively correlated with effector cell activation.
• Multivariable models indicate that IgE diversity to peanut allergens and peanut IgE SA have the greatest influence on effector cell activation.

## Figures and Tables

**Fig E4:**
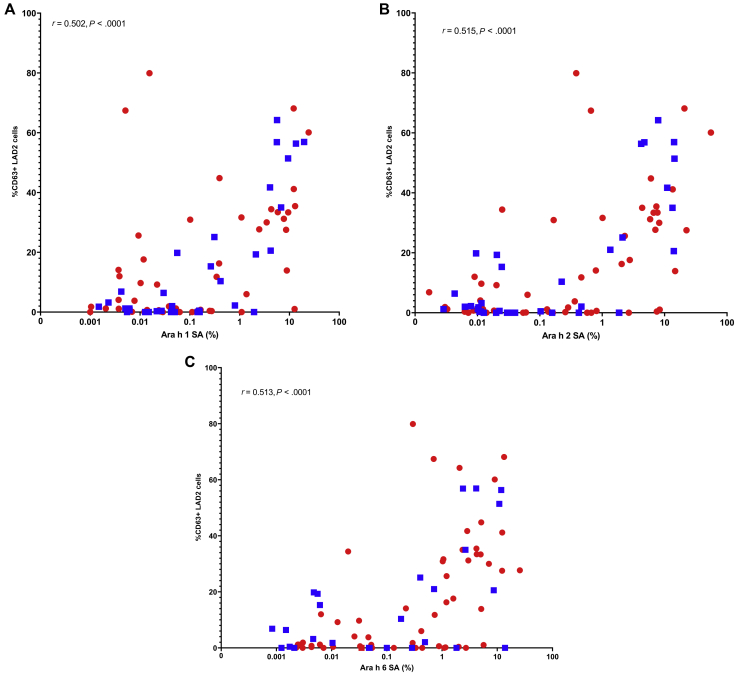
Spearman correlation between (**A**) Ara h 1, (**B**) Ara h 2, (**C**) Ara h 6 IgE SA and mast cell activation. %CD63^+^ LAD 2 cells represent cell activation following the MAT. Correlation reported across peanut-sensitized population (n = 90), with PA individuals (red) and PS individuals (blue). *R* values have been displayed on the figure and in Table II.

**Table E1:** Demographic/clinical features and serum sIgE levels of the study population, used explicitly in avidity assays (n = 60)

Feature	PA (n = 36)	PS (n = 24)	*P* value∗
Age (y)	8.37 (2.06-16.27)	6.05 (0.52-13.71)	.0155
Sex: male	24 (66.67)	15 (62.5)	—
SPT to peanut (mm)	10 (1-34)	3 (0-9)∗	<.0001
tIgE (kU_A_/L)	518 (19-3550)	547 (28-6036)	.5845
sIgE to peanut (kU_A_/L)	31.95 (1.2-568)	5.195 (0.99-20.9)	.0005
sIgE to Ara h 1 (kU_A_/L)	8.92 (0-199)	0.16 (0-11.4)	.0099
sIgE to Ara h 2 (kU_A_/L)	12.3375 (0.05-278)	0.07 (0-0.52)	<.0001
sIgE to Ara h 3 (kU_A_/L)	1.475 (0-89.6)	0.07 (0.02-7.28)	.0115
sIgE to Ara h 6 (kU_A_/L)	10.17 (0-155)	0.03 (0-0.49)	<.0001
sIgE to Ara h 8 (kU_A_/L)	0.215 (0-185)	0.11 (0-88.6)	.7787
sIgE to Ara h 9 (kU_A_/L)	0.04 (0-8.71)	0.13 (0-13.9)	.0354

Values are expressed as n (%) or median (range). *P* value refers to the comparison between PA and PS patients and was generated using Mann-Whitney *t* tests.

∗ SPT in PS individuals (n = 23).

**Table E2:** Correlation matrix for each characteristic assessed in this study across PA and PS individuals (n = 90), including peanut avidity (n = 60)

Characteristic	Age	MAT (%CD63^+^ LAD2 cells)	Peanut-sIgE	Ara h 1 sIgE	Ara h 2 sIgE	Ara h 3 sIgE	Ara h 6 sIgE	Ara h 8 sIgE	Ara h 9 sIgE	Peanut SA	Ara h 1 SA	Ara h 2 SA	Ara h 3 SA	Ara h 6 SA	Ara h 8 SA	Ara h 9 SA	WD	PD	Peanut avidity
Age	1.000	0.357	0.288	0.116	0.431	0.192	0.345	0.425	−0.038	0.069	−0.024	0.311	−0.053	0.239	0.265	−0.269	0.370	0.363	0.249
MAT (%CD63^+^ LAD2 cells)	0.357	1.000	0.787	0.621	0.666	0.613	0.658	0.383	0.219	0.659	0.502	0.515	0.418	0.513	0.146	−0.008	0.494	0.649	0.236
Peanut-sIgE	0.288	0.787	1.000	0.820	0.692	0.865	0.670	0.438	0.416	0.707	0.584	0.448	0.547	0.444	0.046	0.006	0.580	0.645	−0.020
Ara h 1 sIgE	0.116	0.621	0.820	1.000	0.567	0.874	0.535	0.126	0.386	0.623	0.859	0.418	0.677	0.384	−0.214	0.068	0.348	0.551	0.159
Ara h 2 sIgE	0.431	0.666	0.692	0.567	1.000	0.582	0.880	0.268	0.066	0.599	0.473	0.841	0.462	0.736	−0.002	−0.189	0.351	0.809	0.490
Ara h 3 sIgE	0.192	0.613	0.865	0.874	0.582	1.000	0.527	0.270	0.426	0.589	0.666	0.353	0.718	0.319	−0.118	−0.001	0.430	0.547	0.021
Ara h 6 sIgE	0.345	0.658	0.670	0.535	0.880	0.527	1.000	0.242	0.020	0.616	0.479	0.790	0.474	0.891	0.021	−0.147	0.296	0.832	0.470
Ara h 8 sIgE	0.425	0.383	0.438	0.126	0.268	0.270	0.242	1.000	0.442	−0.001	−0.164	−0.036	−0.160	−0.017	0.779	0.101	0.842	0.216	−0.106
Ara h 9 sIgE	−0.038	0.219	0.416	0.386	0.066	0.426	0.020	0.442	1.000	−0.033	0.089	−0.235	0.030	−0.231	0.138	0.692	0.490	0.075	−0.213
Peanut SA	0.069	0.659	0.707	0.623	0.599	0.589	0.616	−0.001	−0.033	1.000	0.749	0.723	0.780	0.687	0.006	0.025	0.135	0.628	0.166
Ara h 1 SA	−0.024	0.502	0.584	0.859	0.473	0.666	0.479	−0.164	0.089	0.749	1.000	0.579	0.834	0.539	−0.211	0.131	0.047	0.512	0.210
Ara h 2 SA	0.311	0.515	0.448	0.418	0.841	0.353	0.790	−0.036	−0.235	0.723	0.579	1.000	0.586	0.871	−0.012	−0.141	0.017	0.736	0.491
Ara h 3 SA	−0.053	0.418	0.547	0.677	0.462	0.718	0.474	−0.160	0.030	0.780	0.834	0.586	1.000	0.545	−0.154	0.088	−0.031	0.517	0.067
Ara h 6 SA	0.239	0.513	0.444	0.384	0.736	0.319	0.891	−0.017	−0.231	0.687	0.539	0.871	0.545	1.000	0.007	−0.117	0.014	0.744	0.493
Ara h 8 SA	0.265	0.146	0.046	−0.214	−0.002	−0.118	0.021	0.779	0.138	0.006	−0.211	−0.012	−0.154	0.007	1.000	0.237	0.537	0.015	−0.061
Ara h 9 SA	−0.269	−0.008	0.006	0.068	−0.189	−0.001	−0.147	0.101	0.692	0.025	0.131	−0.141	0.088	−0.117	0.237	1.000	0.123	−0.083	−0.094
WD	0.370	0.494	0.580	0.348	0.351	0.430	0.296	0.842	0.490	0.135	0.047	0.017	−0.031	0.014	0.537	0.123	1.000	0.285	−0.239
PD	0.363	0.649	0.645	0.551	0.809	0.547	0.832	0.216	0.075	0.628	0.512	0.736	0.517	0.744	0.015	−0.083	0.285	1.000	0.414
Peanut avidity	0.249	0.236	−0.020	0.159	0.490	0.021	0.470	−0.106	−0.213	0.166	0.210	0.491	0.067	0.493	−0.061	−0.094	−0.239	0.414	1.000

*R* values from Spearman correlation have been reported here; for corresponding *P* values, see Table E3.

**Table E3:** Correlation matrix for each characteristic assessed in this study across PA and PS individuals (n = 90), including peanut avidity (n = 60)

Characteristic	Age	MAT (%CD63^+^ LAD2 cells)	Peanut-sIgE	Ara h 1 sIgE	Ara h 2 sIgE	Ara h 3 sIgE	Ara h 6 sIgE	Ara h 8 sIgE	Ara h 9 sIgE	Peanut SA	Ara h 1 SA	Ara h 2 SA	Ara h 3 SA	Ara h 6 SA	Ara h 8 SA	Ara h 9 SA	WD	PD	Peanut avidity
Age		.00056	.00596	.27444	.00002	.06955	.00086	.00003	.72318	.51760	.82589	.00283	.61999	.02349	.01169	.01021	.00034	.00044	.05503
MAT (%CD63^+^ LAD2 cells)	.00056		.00000	.00000	.00000	.00000	.00000	.00019	.03853	.00000	.00000	.00000	.00004	.00000	.16917	.94174	.00000	.00000	.06897
Peanut-sIgE	.00596	.00000		.00000	.00000	.00000	.00000	.00002	.00004	.00000	.00000	.00001	.00000	.00001	.66737	.95223	.00000	.00000	.87807
Ara h 1 sIgE	.27444	.00000	.00000		.00000	.00000	.00000	.23674	.00017	.00000	.00000	.00004	.00000	.00019	.04291	.52120	.00078	.00000	.22635
Ara h 2 sIgE	.00002	.00000	.00000	.00000		.00000	.00000	.01059	.53394	.00000	.00000	.00000	.00000	.00000	.98654	.07482	.00069	.00000	.00007
Ara h 3 sIgE	.06955	.00000	.00000	.00000	.00000		.00000	.01007	.00003	.00000	.00000	.00064	.00000	.00220	.26768	.99553	.00002	.00000	.87582
Ara h 6 sIgE	.00086	.00000	.00000	.00000	.00000	.00000		.02166	.84925	.00000	.00000	.00000	.00000	.00000	.84575	.16804	.00469	.00000	.00015
Ara h 8 sIgE	.00003	.00019	.00002	.23674	.01059	.01007	.02166		.00001	.98997	.12195	.73753	.13151	.87723	.00000	.34142	.00000	.04055	.42142
Ara h 9 sIgE	.72318	.03853	.00004	.00017	.53394	.00003	.84925	.00001		.75874	.40462	.02567	.78122	.02819	.19307	.00000	.00000	.47942	.10218
Peanut SA	.51760	.00000	.00000	.00000	.00000	.00000	.00000	.98997	.75874		.00000	.00000	.00000	.00000	.95836	.81483	.20348	.00000	.20589
Ara h 1 SA	.82589	.00000	.00000	.00000	.00000	.00000	.00000	.12195	.40462	.00000		.00000	.00000	.00000	.04589	.21951	.66325	.00000	.10709
Ara h 2 SA	.00283	.00000	.00001	.00004	.00000	.00064	.00000	.73753	.02567	.00000	.00000		.00000	.00000	.90742	.18384	.87080	.00000	.00007
Ara h 3 SA	.61999	.00004	.00000	.00000	.00000	.00000	.00000	.13151	.78122	.00000	.00000	.00000		.00000	.14827	.41057	.77012	.00000	.60884
Ara h 6 SA	.02349	.00000	.00001	.00019	.00000	.00220	.00000	.87723	.02819	.00000	.00000	.00000	.00000		.94563	.27125	.89866	.00000	.00006
Ara h 8 SA	.01169	.16917	.66737	.04291	.98654	.26768	.84575	.00000	.19307	.95836	.04589	.90742	.14827	.94563		.02439	.00000	.88657	.64115
Ara h 9 SA	.01021	.94174	.95223	.52120	.07482	.99553	.16804	.34142	.00000	.81483	.21951	.18384	.41057	.27125	.02439		.24687	.43530	.47294
WD	.00034	.00000	.00000	.00078	.00069	.00002	.00469	.00000	.00000	.20348	.66325	.87080	.77012	.89866	.00000	.24687		.00638	.06595
PD	.00044	.00000	.00000	.00000	.00000	.00000	.00000	.04055	.47942	.00000	.00000	.00000	.00000	.00000	.88657	.43530	.00638		.00100
Avidity	.05503	.06897	.87807	.22635	.00007	.87582	.00015	.42142	.10218	.20589	.10709	.00007	.60884	.00006	.64115	.47294	.06595	.00100	

*P* values from Spearman correlation have been reported here; for corresponding *R* values, see Table E2.

**Table E4:** Diagnostic performance of each characteristic in the determination of peanut allergy

Characteristic	AUC	Lower bound (95% CI)	Upper bound (95% CI)	Optimal Cutoff	Sensitivity (%)	Specificity (%)
Peanut-sIgE (kU_A_/L)	0.752	0.658	0.845	21.25	32.29	93.10
Ara h 1 sIgE (kU_A_/L)	0.668	0.559	0.777	2.68	32.86	90.00
Ara h 2 sIgE (kU_A_/L)	0.916	0.857	0.975	0.28	82.00	94.00
Ara h 3 sIgE (kU_A_/L)	0.672	0.563	0.780	1.10	33.33	87.10
Ara h 6 sIgE (kU_A_/L)	0.908	0.846	0.970	0.32	82.00	90.00
Ara h 8 sIgE (kU_A_/L)	0.624	0.511	0.737	0.02	60.00	31.43
Ara h 9 sIgE (kU_A_/L)	0.468	0.354	0.581	0.86	14.58	82.69
Peanut S A (%)	0.862	0.793	0.932	1.79	76.00	80.00
Ara h 1 S A (%)	0.716	0.613	0.820	2.29	44.00	100.00
Ara h 2 S A (%)	0.933	0.885	0.981	0.12	92.00	84.00
Ara h 3 S A (%)	0.738	0.639	0.837	0.05	64.00	80.00
Ara h 6 S A (%)	0.930	0.878	0.981	0.29	82.00	92.00
Ara h 8 S A (%)	0.618	0.507	0.730	0.043	54.00	72.00
Ara h 9 S A (%)	0.465	0.351	0.578	0.015	40.00	62.00
PD	0.898	0.831	0.964	0.48	84.00	88.00
WD	0.628	0.517	0.739	1.58	74.00	56.00
Avidity (%inhibition)∗	0.795	0.681	0.910	37.23	61.10	87.50

The AUCs, respective 95% CIs, optimal cutoffs, sensitivity, and specificity have been reported alongside each characteristic.

∗ Avidity cohort, n = 60.

**Table E5:** Pairwise comparison of ROC analyses in the same sample cohort (n = 100)

Characteristic comparisons	Diagnostic performance (ROC analyses)						Pairwise comparison 8of AUCs (*P* value)
	AUC	Lower bound (95% CI)	Upper bound (95% CI)	AUC	Lower bound (95% CI)	Upper bound (95% CI)	
Peanut-sIgE (kU_A_/L)-Peanut SA (%)	0.752	0.658	0.845	0.862	0.793	0.932	.0038
Ara h 1 sIgE (kU_A_/L)-Ara h 1 SA (%)	0.668	0.559	0.777	0.716	0.613	0.820	.0898
Ara h 2 sIgE (kU_A_/L)-Ara h 2 SA (%)	0.916	0.857	0.975	0.933	0.885	0.981	.5415
Ara h 3 sIgE (kU_A_/L)-Ara h 3 SA (%)	0.672	0.563	0.780	0.738	0.639	0.837	.0664
Ara h 6 sIgE (kU_A_/L)-Ara h 6 SA (%)	0.908	0.846	0.970	0.930	0.878	0.981	.2420
Ara h 8 sIgE (kU_A_/L)-Ara h 8 SA (%)	0.624	0.511	0.737	0.618	0.507	0.730	.8743
Ara h 9 sIgE (kU_A_/L)-Ara h 9 SA (%)	0.468	0.354	0.581	0.465	0.351	0.578	.9552
WD-PD	0.628	0.517	0.739	0.898	0.831	0.964	<.0001

Comparison of AUC paired data ROC curves as described by deLong et al.^E1^

**Table E6:** Diagnostic performance of selected characteristics in the determination of peanut allergy, in reduced study cohort used for avidity assays, as governed by the areas under the ROC curve

Characteristic	AUC	Lower bound (95% CI)	Upper bound (95% CI)
sIgE to peanut (kU_A_/L)	0.760	0.641	0.880
sIgE to Ara h 2 (kU_A_/L)	0.944	0.885	1.000
sIgE to Ara h 6 (kU_A_/L)	0.947	0.889	1.000
Peanut SA (%)	0.843	0.746	0.939
Ara h 2 SA (%)	0.970	0.933	1.000
Ara h 6 SA (%)	0.954	0.899	1.000
PD	0.931	0.866	0.995
WD	0.505	0.348	0.661
Avidity (%inhibition)	0.795	0.681	0.910

The AUCs and their respective 95% CIs were generated in the cohort described in Table E1 and allows for comparison of diagnostic performance in the reduced study cohort.

**Table E7:** Simple multivariable logistic regression models (n = 90), in which combinations of functional IgE characteristics were used to predict %CD63^+^ LAD2 cell activation

Supplementary model no.	Characteristic	Unstandardized *β*-coefficient	Coefficient SE	Standardized *β*-coefficient	*P* value	*R*^2^ value	VIF
S1	PD	22.005	3.196	0.588	<.0001	0.411	1.101
	WD	3.894	2.143	0.155	.073		1.101
S2	Peanut-sIgE	0.050	0.016	0.279	.003	0.454	1.322
	Peanut SA	0.765	0.142	0.491	<.0001		1.322
S3	PD	15.752	3.260	0.421	<.0001	0.523	1.381
	Peanut SA	0.635	0.136	0.407	<.0001		1.381

Unstandardized and standardized *β*-coefficients are reported alongside significance and variance inflation factor (VIF). VIF >10 was used to indicate any collinearity.

**Table E8:** Simple linear regression models, in which functional IgE characteristics were used to predict %CD63^+^ basophil activation

Characteristic	Unstandardized *β*-coefficient	Coefficient SE	Standardized *β*- coefficient	*P* value	R^2^ value
Peanut-sIgE	0.151	0.019	0.737	<.0001	0.543
Ara h 1 sIgE	0.392	0.067	0.632	<.0001	0.399
Ara h 2 sIgE	0.338	0.041	0.756	<.0001	0.564
Ara h 3 sIgE	0.875	0.130	0.682	<.0001	0.465
Ara h 6 sIgE	0.674	0.091	0.717	<.0001	0.514
Ara h 8 sIgE	−0.232	0.280	−0.114	.410	0.130
Ara h 9 sIgE	−0.011	1.783	−0.001	.995	0.000
Peanut SA	1.434	0.169	0.762	<.0001	0.581
Ara h 1 SA	3.772	0.584	0.584	<.0001	0.445
Ara h 2 SA	2.119	0.322	0.674	<.0001	0.455
Ara h 3 SA	11.606	1.555	0.719	<.0001	0.517
Ara h 6 SA	5.681	0.908	0.655	<.0001	0.429
Ara h 8 SA	−1.692	2.109	−0.111	.426	0.012
Ara h 9 SA	−2.567	5.088	−0.070	.616	0.005
WD	8.378	4.315	0.260	.058	0.068
PD	40.432	3.578	0.843	<.0001	0.711
Avidity	0.635	0.378	0.281	.102	0.079

Unstandardized and standardized *β*-coefficients are reported alongside significance (n = 54).

**Table E9:** Simple multivariable linear regression models, in which combinations of functional IgE characteristics were used to predict %CD63^+^ basophil activation (n = 54)

Model no.	Characteristic	Unstandardized *β*-coefficient	Coefficient SE	Standardized *β*-coefficient	*P* value	R^2^ value	VIF
S4∗	Peanut-sIgE	0.043	0.025	0.213	.098	0.765	2.045
	PD	38.851	7.119	0.751	<.0001		2.50
	Avidity	−0.230	0.227	−0.102	.317		1.326
S5∗	Peanut SA	0.616	0.187	0.335	.002	0.809	1.690
	PD	35.673	5.787	0.690	<.0001		2.037
	Avidity	−0.243	0.200	−0.108	.234		1.280
S6∗	Peanut-sIgE	0.138	0.025	0.688	<.0001	0.540	1.041
	WD	1.372	6.740	0.025	.840		1.007
	Avidity	0.352	0.280	0.156	.219		1.037
S7∗	Peanut SA	1.374	0.214	0.748	<.0001	0.607	1.071
	WD	9.911	6.270	0.180	.124		1.021
	Avidity	0.280	0.260	0.124	.290		1.049
S8	PD	41.936	3.885	0.874	<.0001	0.716	1.179
	WD	−2.594	2.610	−0.81	.325		1.179
S9	Peanut-sIgE	0.098	0.016	0.482	<.0001	0.760	1.299
	Peanut SA	0.998	0.147	0.531	<.0001		1.299
S10	PD	28.841	3.927	0.601	<.0001	0.796	1.680
	Peanut SA	0.714	0.154	0.380	<.0001		1.680

Unstandardized and standardized *β*-coefficients are reported alongside significance and variance inflation factor (VIF). VIF >10 was used to indicate any collinearity. For models containing avidity as an input characteristic, n = 35 is observed.

∗ For models that include avidity analyses, n = 35.
